# Singing as a health-promoting activity in elderly care: a qualitative, longitudinal study in Norway

**DOI:** 10.1177/1744987120917430

**Published:** 2020-06-08

**Authors:** Kari Bjerke Batt-Rawden, Kristi Stedje

**Affiliations:** Associate Professor/Sociologist, Department of Health Sciences Gjøvik, Norwegian University of Science and Technology, Norway; Music Therapist, Department of Music and Health, University College of Music, Norway

**Keywords:** care of the older person, care staff education, health promotion, singing

## Abstract

**Background:**

The current standards of care of the older person recommend employing non-pharmacological approaches to challenges, including safe approaches to managing pain and stress, enhancing symptom relief, and fostering independent lifestyles with the highest quality of life possible. More research is needed to enable nurses and other medical staff to use singing and music-based interventions, to access singing-based programmes, and promote a greater use of choirs in nursing homes. A solid basis of positive experiences and feedback through evidence in practice is required to help promote support for such activities.

**Aims:**

To identify, explore and describe experiences, attitudes, beliefs, issues, processes and changes among nurses, carers and leaders in reference to implementation of the educational programme ‘Singing Nursing Homes’, Norway. To increase knowledge and understanding of how an educational song programme could become an integral part of nursing practice and quality of care in nursing and care homes, and identify why this would be constructive. Since its inception in 2015, ‘Singing Norway’ wanted to offer a professional, evidence-based programme for nursing homes throughout the country.

**Methods:**

A longitudinal, qualitative and explorative approach. In-depth interviews and focus group interviews of female employees (*n* = 19) from three nursing homes in Norway, 2018.

**Results:**

Nursing home employees perceived singing to have potential benefits for their patients, such as reducing uneasiness, increased comfort, well-being and joy, improved sleep, and believed singing had the potential to reduce the need for medication and prevent accidents among their patients. By facilitating opportunities for learning and practice, staff in nursing homes were able to use singing as part of their ‘art of caring’, enhancing environmental care for the older person. Singing was found to have positive effects for the patients, their relatives and the staff, which improved the psychosocial working climate overall.

**Conclusions:**

Singing interventions could be a vital component for the enhancement of health, well-being and quality of life for the patients and staff in nursing homes.

## Introduction

### Singing and musicking as a health-promoting activity in caring for the older person

Research into singing has shown widespread beneficial effects on health and well-being (Clift and Morrison; 2011; Gunter, 2004; Williams et al., 2018), and could be described as a salutogenetic activity (Antonovsky, 1996). As the topic of singing and well-being has attracted increased attention since the late 1990s, a number of literature reviews have appeared to critique and synthesise existing evidence (Clift et al., 2016). There are increasing numbers of reviews focused on the health and well-being of older people, and on musical interventions that address the well-being and care of people with dementia (Van der Steen et al., 2018). The current standards of care of the older person recommend non-pharmacological approaches to current challenges, including safe approaches to managing pain and stress, enhancing symptom relief, and fostering independent lifestyles with the highest quality of life possible (Magee and Davidson, 2004; Vink and Hanser, 2018). Complementary and alternative medicine (CAM) may provide unique opportunities to improve patient health and manage pain through non-pharmacological treatment and care with minimal adverse effects (Clift et al., 2010).

In multiple studies, music has been shown to benefit a variety of patients by helping reduce depression, anxiety and pain levels, while also being cost-effective (Chan et al., 2011; Erkkilä et al., 2011; Kneafsey, 1997; Mathur et al., 2008; Van der Steen et al., 2018; Wang, 2010). Kneafsey (1997) focused on music therapy and care of the older person with debilitating illnesses such as Parkinson’s disease and dementia. Meaningful social activities, tailored to the older individual’s abilities, preferences and needs should be considered when aiming to improve mental health among older people (Forsman et al., 2011). Music-based environmental therapy has a positive effect on well-being and drug consumption, and music has been found to have a particularly positive effect in the face of unease and depression in patients with dementia (Boal-Palheiros, 2017; Magee and Davidson, 2004; Myskja, 2011; Osman et al., 2014; Vink, 2001; Wigram et al., 2002).

Music’s beneficial effects seem to be created in and through health musicking (Stige, 2005), which can be understood as any use of musical experiences to regulate emotional or relational states or to promote well-being, be it therapeutic or not, professionally assisted or self-made (Bonde, 2011: 140). It must be stressed that health musicking is not limited to a professional therapeutic context, but can be observed in any social or individual practice where people use music experiences to create meaning and coherence in states and times of adversity (Bonde, 2011: 135). Ansdell (2004: 83, 86) adds the concept of ‘musical communitas’ to describe a common shared world of time, space, gesture and energy that allows both diversity and unity. How the ‘music of everyday life’ can afford clients and communities just what they need.

### Gaps in Knowledge

New regulations (Norwegian Ministry of Health and Care Services, 2013, 2018, 2019) have called for music to be employed as an alternative or complementary approach to treatment, potentially lessening the need for and use of medication. However, educating the public as well as health professionals is crucial to advocate for CAM therapies in nursing care plans, as identified in a recent study depicting systematic use of music as an environmental intervention to improve quality of care in nursing homes (Batt-Rawden and Storlien, 2019). However, more research is needed to enable nurses and other medical staff to use the arts as an intervention, including music-based interventions, singing-based programmes and greater use of choirs in nursing homes (Clift et al., 2016).

Clark and Harding’s (2012) systematic review of the psychosocial outcomes of singing interventions concluded that more qualitative studies were needed. Music-based interventions seem to be inconsistent, and practitioners vary in their professional training and preparation for implementing music-based clinical strategies (Magee and Davidson, 2004). Most studies on intergenerational relationships and their impact on health, well-being and learning through singing involve interventional programmes (David et al., 2018; Beynon and Lang, 2018; Heydon et al., 2017). Moreover, despite considerable research and evaluation of the value of singing in nursing homes, a solid basis of positive experiences and feedback through evidence in practice (EIP) is still required (Clift et al., 2016; Williams et al., 2018).

### Purpose and Aim

The main purpose of this paper is to increase knowledge and understanding of how an educational song programme could become an integral part of nursing practice and implemented in daily care in nursing homes, and furthermore, evidence why this would be constructive.

The aim of the research was to explore, describe and identify experiences, attitudes, beliefs, issues, processes and changes among nurses, carers, medical staff and leaders in reference to implementation of the educational programme ‘Singing Nursing Homes’, Norway.

## Methods

### A qualitative, longitudinal study

This longitudinal study had a qualitative and explorative approach. A qualitative methodology is well-suited to gaining insights into participant experiences, thoughts and feelings. It enables researchers to answer the ‘how’ and ‘why’ type questions, while taking into consideration how a phenomenon is influenced by the context within which it is situated (Charmaz, 2003; Dawn and Spencer, 2003; Murray, 2003; Silverman, 2011). Since this study employed a qualitative approach, theories and conceptual frameworks are only presented to underpin knowledge in the current field. We have tried to highlight our results and major findings in relation to previous research and theories that seemed appropriate.

Constructivist grounded theory was chosen as the methodology to explore and describe processes around the activity of singing as they emerged from the data. The inductive nature of grounded theory methods assumes an open, flexible approach. It allowed the iterative shaping of methodological strategies while engaged in the research, rather than having them rigidly fixed before beginning the data collection. The comparative methods that are key to grounded theory analysis, enabled the whole research process to be interactive and iterative so that new, and potentially useful theories could be generated from our data (Charmaz, 2017). While grounded theory does not specify suitable data collection methods, it can enrich our understanding of singing and music’s mechanisms of operation in naturalistic settings and in ways that are derived from the meaning systems of people (participants) themselves. In this way it provides a means for exploring correlations between singing, music and health promotion as found through more quantitative and experimental work (e.g. in the realm of music and medicine) by helping to uncover the ways in which singing and music’s health benefits are understood and experienced by the actors themselves (Charmaz, 2003).

### Data Collection and Analysis

The sample consisted of only women (*n* = 19), and was strategically and conveniently selected, comprising nurses, leaders and carers from three nursing homes in the county of Akershus, Norway. The nursing homes were given fictive names: the Dandelions (*n* = 6), the Daisies (*n* = 8) and the Edelweiss (*n* = 5). Passive observations lasting 2 days in two departments (totalling to 4 days) of the nursing homes were executed in order to observe the singing programme in practice, hence two singalongs and one concert took place at all three homes. These visits took place in the same period as the interviews. An open-ended guide was designed to give both structure and flexibility to enable themes to emerge from the informants’ own accounts. Individual interviews and focus group interviews were conducted in two periods to gain a more thorough understanding of the main purpose and aims of the research, as outlined. Data was collected from March to April and from August to September 2018. During this period, both in-depth interviews and focus group interviews were conducted at each of the three nursing homes. Participant numbers in the focus group interviews varied from five to eight participants.

In the first period, individual in-depth interviews followed by focus group interviews were conducted during the same week. The same people participated in both interviews. Since this was a longitudinal study from March to September, participants were followed up over time. During the second period only focus group interviews were conducted at each institution. This second round focused on the same issues, and whether anything new had occurred in reference to processes and changes from the first period.

The data analysis was consistent with the constructivist grounded theory approach. From the start, we attempted to conceptualise what was being described in the interviews and focus groups based on inductive procedures for obtaining emergent themes (Charmaz, 2003; Murray, 2003). Data analysis was carried out using line-by-line coding for identifying concepts, detecting themes, defining events, actions and situations. Line-by-line coding helped to review the data in a new light: what is going on? What are the participants doing? What is the person saying? What do these actions and statements mean? (Charmaz, 2003). The next step was more selective and focused on coding for verifying relationships between concepts. We compared people in terms of factors such as their beliefs, opinions, situations, actions, accounts or experiences. In the focused coding, we took a limited number of line-by-line codes and applied them to a large amount of data.

In doing this, we tried to stay as close to the data as possible (Charmaz, 2003) through labelling recurrent themes that reflected the substance of the data. Furthermore, some of these themes were collated into general themes, which emerged by developing the main concepts induced from recurrent themes to build a grounded theory. All participant quotes were coded (line-by-line and focus coding) and incorporated into separate files as ‘categories’. Line-by-line coding gave us leads to pursue in the interviews that followed, or was used as a method to revisit previous interviews. Hence our data analysis became more focused on analysing connections, and developing categories and concepts. To analyse patterns and tendencies, we used different colours for significant concepts, keywords, emergent themes, and important phrases and compared these to strengthen the reliability and trustworthiness of the study (Charmaz, 2017). In this way, we obtained an overall impression of our data, what they consisted of and identified pertinent factors, contributing to greater knowledge and a deeper understanding throughout the processes of analysis and interpretation.

Recurrent themes (developed into concepts and categories) can be described as themes that both occur several times in relation to one participant and/or among the sample as a whole. For example, ‘how singing works’ or ‘does it work?’ or ‘affects you, patients and/or the environment’, created codes such as ‘vitality’, ‘joy’, ‘energy’, ‘better humour’ and sense of ‘belonging’, ‘connectedness’, ‘calmness’, ‘hope’, ‘memories’, ‘good mood’, ‘better sleep’, ‘less medication’, ‘passive’, ‘active’, ‘move’, and ‘fewer accidents’. These were developed into subcategories: ‘well-being’, ‘health promoting’, beneficial’, ‘psychosocial working climate’, and subsequently labelled (conceptualised) as ‘the beneficial outcome of the singing programme’, ‘the staff’s experiences of singing for and with the patients’ and ‘the impact of singing on staff’s psychosocial working climate’. These were systematically compared with other identified code categories. This was further developed into sensitising concepts such as ‘the salutogenetic impact of the art of singing in nursing homes’ and ‘the value of informal learning of singing for self and others as caregivers’.

### The programme: Singing Nursing Homes

Since its inception in 2015, Singing as caring for the older person, a part of Singing Norway wanted to offer a professional, evidence-based programme for institutions throughout the country, to promote knowledge about and implementation of singing as a health promoting activity and job-creating measures in everyday life. In the pilot period 2015–2018, a total of 26 institutions/nursing homes followed the programme, and received courses, supervision and material administered by the operator organisation, The Folk Academy, and sub-operator, Akershus Music Council, Norway.

In Singing Nursing Homes song is implemented on three levels: Singing as caring of the older person gives the nursing homes material, guidance and courses that help them achieve the goal of implementing song. Seven course modules were developed, and the institutions put together their course package in collaboration with Singing as caring of the older person based on their specific wishes and needs. The academic and theoretical basis is rooted in humanistic music therapy (McDermott et al., 2018; Ruud, 2010), resource-oriented thinking (Rolvsjord, 2010), music as affect regulation (Ridder, 2017), as well as brain research and neurology (Brean and Skeie, 2019) and music from a public health perspective (Bonde and Theorell, 2018).

This study is the first formal research on the implementation of the Singing Nursing Homes programme.

## Ethics

All participants were given written information about the project prior to data collection. All participants had to sign written consent. It was emphasised that participation was voluntary, with the right to withdraw from participation at any point during the study. They willingly consented to participate, and there were no problems of access to the field of study. The interviews were recorded and transcribed verbatim. This project was declared not subject to notification in March 2018 by the Data Protection Official for Research, Norway (www.nsd.uib.no/personvernombud/en/notify/index.html), since all electronic data from the entire research process were anonymised. In addition, no sensitive data could be linked to directly identifiable personal data or indirectly through a combination of background information, such as place of residence or institutional affiliation, combined with data on age, gender, occupation, or university. In carrying out interviews, the only personal data documented were in the form of notes; these notes, including recordings, were redacted to ensure that no names or personal background information was registered in the data. Respecting the needs and wants of the participants was paramount throughout the study. An issue with small studies is how to protect confidentiality, as people may be identified by their experiences or expressions, thus, we were sensitive to this. Consent forms were kept secure, separate from transcribed data.

## Results

### Overview

The findings indicate that nursing home employees perceived singing to have potential benefits for their patients, such as reduced uneasiness, increased comfort, well-being, joy, improved sleep, and to reduce the need for medication, even potentially reducing the risk of accidents among their patients. By facilitating opportunities for effective learning and practice, staff in nursing homes could use singing as part of their art of caring, enhancing environmental care for the older person. Singing has positive effects for patients, their relatives and the staff, which often benefits the psychosocial working climate. Singing interventions could be a vital component of the enhancement of health, well-being, and quality of life for patients and staff in nursing homes. To achieve success and lessen barriers in the implementation of singing-based interventions in nursing homes, there needs to be a raised level of commitment, and changes in beliefs and attitudes surrounding what constitutes quality of care and nursing, hence why engagement with and encouragement from management is important.

### The beneficial outcomes of the singing programme

At all three nursing homes, the staff had a lot of experience with dementia, and were able to observe the effects of the musicking activities, be it singing, listening to personal music on CDs or attending concerts and singalongs. Several staff members expressed how singing in particular seemed to be important for patients with dementia, voicing that ‘there is something about getting the songs into the whole image of what health is and can be’. By observing changes in posture, mood, facial expressions like smiling and laughing, these good, warm feelings seemed to last for a long time after these sessions:

We have a patient with dementia with us. She doesn’t have much language, but she is quite good at singing … she loves to sing, she can have a lot of songs and we encourage her, and tell her how good she is. It is quite clear that she is very proud about her singing. I have made a children’s songbook for her, and I have observed that she is still singing after I have left (nurse).

Several of the staff claimed they had observed many different ways singing songs and listening to music had helped in various situations for ‘mood and psyche and everything’. Several of the nurses reported how a number of patients changed dramatically from being passive to actively showing renewed energy, vitality and good mood after a concert or a singalong. Some patients also started to move, and moving to music was perceived as ‘fun’:

I remember a patient, she was very uneasy and sat down on the floor for one hour, then we started singing a little for her and then she got up and sang (carer).

The fact that these singing activities, like lullabies, singalongs or one-to-one humming reduced uneasiness, increased comfort and improved sleep was documented by many of the nurses. A typical statement from a nurse also highlights the ‘magic’ of mood change seen among several patients after a singing event:

After we are finished, their faces light up, and they are uplifted. It is no doubt how song and music can lead to something completely magical, a kind of exalted mood that amazed many here. The older generation also seems to remember the lyrics from the old songs. For example, although some patients could have stroke or Parkinson’s, they could still sing (nurse).

Some nurses were quite astonished to see how memories through songs affected the patients, and how singing could produce such strong feelings and emotions. In some situations, it was tear-jerking, precisely because it brought out so much emotion; as one nurse put it,

A patient said to me that she is usually crying when she hears some particular songs, but she said there is nothing wrong with that, so, you see, it arouses something in me, and it’s just good. The music goes deep in (nurse).

### The staff’s experiences of singing for and with the patients

The experience of observing the power of singing was followed by the desire to tell others about these positive effects. Singing songs with and for the patients could also reduce medication. It was pointed out how their concern for over-medication in the nursing homes resulted in adverse side-effects form medicines. At one institution they believed there was a connection between frequent singing practice and the minor use of medication; as one nurse said,

We sing so much here, so [laughing], there are not so many people who use medication for anxiety, depression or disorderly behaviour.

The fact that singing songs could actually have a preventive effect against accidents in the departments was an interesting finding to emerge. The singing sessions or singalongs in the evening resulted in calmer, less anxious and more relaxed patients, which demonstrates how singing influences behaviour. Patients sat quietly reflecting, even humming, until they were helped to bed, subsequently sleeping well with less or even no medication.

In my department there could be a lot of turmoil in the evening, but if someone plays or sings in the living room, I think singing may prevent accidents, because they sit there until we come – and then they do not have to break any legs or hips, and they sleep so good and they are so happy. We can see that they have had a nice day and night, you can see it on their body. Singing does create peace, feelings of being safe, and it also prevents accidents and less use of medication or drugs (leader of a nursing home).

Several of the staff described various past experiences of singing. Someone who had been singing for many years, was happy to sing in front of patients, relatives or colleagues. A vital point here is how singing itself should not be associated with assessments of voice quality (‘a good singer’, ‘bad or poor singer’) or a lack of trust in being able to find their own voice (‘not able to sing’, ‘do not dare to sing’). It was not the achievement of singing that was the focus of the educational singing programme here, but to inspire and encourage the nurses to be confident and have trust in their own singing voice for healing purposes and health benefits.

As one nurse said, ‘there is no audition’. Instead there were stories to be told and exchanged, and music and songs played that often related to their own biography and that of the patients’. The art of caring mediated through singing also seems to be connected to the value of sharing memories and associations with the patients. A recurrent theme from all three nursing homes was how the focus on singing had led to greater self-confidence and a sense of mastery of the employee’s own voice. The focus on singing and music also seemed to increase an awareness of music’s use in everyday life:

I sing a lot more one-to-one now [with one patient] and I have become more aware of this [singing], but I have always loved singing. I dare to sing more in other settings, too, so now I have begun in a choir. I have also started to sing more for the family, at birthday parties and so on, so I think I am more confident, you know (carer).

The act of singing for self and others did, however, also create problems for some nurses and carers. Some stated that they had not sung since school. They also held the belief that they would not ‘be able to sing’, especially if their colleagues were listening. Despite the singing programme’s emphasis on ‘finding your own voice’, some nurses claimed that it had been difficult at times to join in with the ones who loved to sing, reporting feeling scared and shy, even hiding:

Maybe I do have a voice, but I think it is embarrassing, but at the same time there are many who are happy to sing (nurse).

An interesting aspect was how some employees changed their attitudes from ‘I can’t sing’ to ‘do it’. By observing their patients’ appreciation of these singing activities, which seemed to be totally independent of the quality of the employee’s singing voice, their confidence increased. As such, putting singing on the agenda in daily practice had contributed to positive changes in bringing out the joy of singing:

I have a colleague who has said for years, I can’t sing, I can’t sing, but I hear she can sing. I think maybe it’s a bit like we’re actually being sent on such a ‘sang [*sic*] as caring of the older person course, and then you have to give something back, as well, because it is something they really focus on here (carer).

### The impact of singing on staff’s psychosocial working climate

Several of the staff stated that singing and music should be seen as a health-promoting resource that should be an obligatory part of nursing education and, hence, practice. A major finding was that all three nursing homes emphasised how singing and musical activities led to a good and thriving working climate. Phrases like ‘good community feeling’, feelings of ‘well-being’, better ‘humour’ and sense of ‘belonging’ among the staff were typical.

When we have music, I can see that the employees are in a very good mood, and we can see and observe this in the patients too (leader of a nursing home).

Quotes such as singing ‘does something with the body’ and that ‘the patients are happy’, ‘it can last for two or three hours afterwards’ and, ‘we have something to talk about’, illustrate the practical in-situ observations of the art of singing in action. All three nursing homes were in agreement about the enthusiasm, positive collaboration and eagerness to disseminate current knowledge of the beneficial aspects of singing:

We had a kick-off meeting when they [representatives from the educational singing programme] had information about the project. We can see that those [nursing homes] who have a responsible person, they have been able to commit much more time to the project (Nurse).

The staff, patients and the relatives seemed to enjoy how singing and musical activities were actively and regularly used in the departments. At one institution they agreed to put singing on the agenda three times a week.

I notice that people are humming more in the hallways, and I hear several of the other nurses say the same. We are always in a good mood after we have had these singing moments (Nurse).

Furthermore, especially in specific caring situations, singing was found to be a pleasant and thriving activity for both nurses, carers and patients, enhancing communication and a sense of well-being. Some called singing a form of ‘musical care’, enhancing quality of care that should not be underrated.

## Discussion

### Strengths and limitations of the study

In qualitative studies, it is important to reflect upon and consider the role of the researcher(s). The ‘observer effect’, that is, the researcher’s gender, age, and personal characteristics, needs to be taken into account (Dawn and Spencer, 2003; Silverman, 2011). However, a researcher is not without history, and needs to be reflexive throughout the research process. In this respect, our personal ‘style’, enthusiasm, and interest when undertaking the fieldwork may have influenced the participants in a positive manner. An aspect worth considering is whether these results would have been comparable if someone else had conducted the research. At the same time, there was an ongoing process of self-reflection during this project in our roles as researchers, which contributed to the trustworthiness and credibility of this study (Charmaz, 2003, 2017; Dawn and Spencer, 2003; Malterud, 2001; Murray, 2003; Silverman, 2011).

In assessing the validity or credibility of the work, we acknowledge the limitation of a small sample from three nursing homes situated in a central region in Norway. However, there are many nursing homes in Norway that have cultural and demographic contexts comparable to this region. This sample is not statistically representative; nevertheless, some tentative, general interpretations might be proposed, that is, feasibly, related projects in the health sector or other nursing homes elsewhere might explore similar implications and inferences.

A feature of a typical cross-sectional study is the fact that it may be difficult to elicit data about a complex topic in one-off interviews at a single point in time, producing fewer opportunities to establish an in-depth rapport and rich descriptions (Morse, 1995). A strength of this study was its longitudinal design, which enabled comparison of participants’ experience and practices at two different points in time, thereby linking these findings to context, situation and meaning. Comparing processes and changes in how participants related to the implementation process, revealed how practices, expectations, and attitudes changed over time (Rokstad, 2012).

We also believe the added value of this two-stage interview process is that it could address researcher bias. Sample and method bias was tackled using triangulation data collection methods (interviews, observation and focus groups) in three nursing homes. Regarding the use of focus group interviews, the interaction among the participants uncovered tacit and experience-based knowledge from the field, raising participants’ awareness and singing as a health promoting activity in elderly care (Silverman, 2011). As such, this type of data collection method was well-suited to discussing the common perceptions and variants of a given topic or issue. Group interviews also provided information through the participants’ shared experiences. Though there is always the possibility that a group may exert influence that inhibits an individual speaking freely, this was not observed to be an issue here. The focus group is also a data collection technique that is distinct from in-depth interviews in that is provides researchers with data that relies upon the interaction of the group members to formulate answers to the researcher’s questions.

One disadvantage of using focus group interviews is that the researcher has less control in the interview setting in relation to individual interviews. Another limitation is that a participant may have strong personal opinions, and thereby dominate and control the other participants who may not, as a result, speak freely or open their minds to the topic (Litosseliti, 2003). As researchers, maintaining a reflexive attitude is important when interpreting and presenting the data. Focus groups require an even greater level of attention from the interviewer, because there are several interviewees participating. In addition to the factors considered in the conduct of in-depth interviews, interviewers conducting focus groups must also attend to the relationships developing between the group members. In focus groups, interviewers should be unobtrusive, draw all interviewees into the discussion by encouraging interaction, and use strategic summarisations of the discussion to help the group refine its thoughts or explanations.

Regarding the one-to-one relationship between an interviewer and the participants, the individual’s experiences in qualitative interviews are represented in dialogue, which formulates the interviewee’s own ‘life world’. Through individual interviews with the participants, we believe we have gained knowledge of the their unique experiences and understanding of this topic. Furthermore, the group member dynamics may have stimulated discussion of topics and issues not necessarily explored in the individual interviews. An action research approach may also have elicited rich data from the processes and changes occurring during the project (Whyte, 1991).

### The salutogenetic impact of the art of singing in nursing homes

Music and singing are effective methods for reducing anxiety levels in older adults, and are generally beneficial to their health, well-being and quality of life (Eells, 2013). In a study by Götell et al. (2009) caring of the older person seemed to be more energised, more cognitively aware, and more responsive to the caregivers if the caregivers were singing and also received responses from the elderly. The fact that singing activities such as lullabies, singalongs and one-to-one humming reduced uneasiness, and increased well-being and comfort has been documented by several authors (Batt-Rawden and Storlien, 2019; Besha, 2015; Clift et al., 2016). These results also support Hammar et al.’s (2010, 2011) finding that negative emotions such as anger, sadness and anxiety seemed to decrease, while positive emotions such as happiness, and the expression of such emotions through smiling, whistling and laughing increased following the introduction of singing. In other words, singing helps participants to achieve happiness, contentment, satisfaction and peace (Lehmberg and Fung, 2010).

Following these arguments, both singing and music interventions could prove effective alternatives to medication such as sleeping pills, which can have side effects for older persons (Smith, 2009; Villarreal et al., 2012). Non-pharmacological interventions like music and singing can reduce the need for medication, indicating they could be considered effective nursing interventions for older persons experiencing, for example, chronic pain (McCaffrey and Freeman, 2003; McCaffrey and Locsin, 2006); there is already evidence that listening to music reduces acute and chronic pain (Kwon et al., 2006; Villarreal et al., 2012).

Several of the staff also reported observing the many ways singing songs and listening to music helped in various situations for ‘mood and psyche and everything’. As described here, singing improved mood, made the patients happier, calmer and more content. Understanding this, music thus becomes an empowering asset that offers listeners a unique sense of personal control over their experiences, which can add to their emotional well-being. Previous research has also shown how singing activities can have an impact on energy levels, improve focus, enhance mood and promote relaxation in older persons with functional disabilities and in their caregivers. (Batt-Rawden and Andersen, 2019; Besha, 2015; Davidson and Almeida, 2014; Götell et al., 2000; Wise et al., 1992).

As noted in this study, singing songs with and for the patients was believed to reduce potential accidents due to patients being calmer and less anxious. As reported, the patients ‘sit there until we come – and they do not have to break any legs or hips’. Our study supports a significant finding by Cohen et al. (2006) and Cohen (2009) that participants in the singing group were less likely to fall during the time of the intervention. According to Clift et al (2016), considering the prevalence of falls among older people and the short- and long-term consequences for their well-being, not to mention the cost to the health service, this finding warrants further exploration, a view we support here. Perhaps the context of residential care, and collating data on falls for residents participating in singing activities compared with those who are not, could serve to test this important finding from Cohen et al. (2006).

### The value of informal learning of singing for self and others as caregivers

As we have seen in this study, the experience of observing the power of singing, led to the desire to tell others about its positive effects, despite a certain amount of employee anxiety about singing at the onset. It seems that the employees could identify what ‘worked’, and learnt how to invoke and empower the ‘charm’ of singing and empower themselves in relation to this. These musical/singing practices do sometimes seem to be tacit, revealed only when the mind is set to think otherwise, for example, the move from ‘I can’t sing’ to ‘I can sing’. As Csikszentmihalyi (1988: 27) asserts: ‘pleasure, power, and participation are some of the basic models on which a self can be built. In consequence, the lay knowledge of the beneficial aspects of singing for care of self and others seemed to be learnt and embodied in the ‘doing’ of these moments. When introduced to musical learning situations, formal or informal, music and singing becomes an arena for the development of self-efficacy and trust (Small, 1998), and the singing mind may have a strong influence on the identity construction for all actors involved. That may be the contextual ‘effect’ of singing and music.

As pointed out by other researchers, training on how to use singing as a tool for nurses and caregivers requires no special equipment other than the singing voice. If singing and its techniques are implicit in our primal method of communication and our emotional connectivity, this could explain why singing more so than music per se, or other art forms, can create such strong effects (Clift et al., 2016). A recurrent theme emerging from all three nursing homes was how the focus on singing had led to greater self-awareness, consciousness and confidence, enhancing a sense of mastery over their voices. On conscious or unconscious levels, people appropriate music on a daily basis to either create, change, maintain or enhance their affect (DeNora, 2000; Saarikallio and Erkkilä, 2007; Skånland, 2012; van Goethem, 2010).

Several of the staff stated that singing should be seen as a health-promoting resource that should enter nursing education. Many also wondered why this subject was not yet part of their education, given the knowledge of musicking and its health benefits. From this viewpoint, learning new practices through empowering, enjoyable rituals, may contribute to self-development (Csikszentmihalyi, 1990) as, for example, learning how to detect valuable moments in life. From this perspective, singing could be a door-opener to an encounter with inner experiences not usually open to conscious articulation (Butterton, 2004).

Training on using singing as a tool for caregivers to interact with elderly persons during daily situations in caregiving, seems to be of significance. Several studies have demonstrated that focused educational interventions have proven to be effective in addressing knowledge, skills and attitude gaps related to EIP in both nurses and nursing students (Stige, 2005; Vink and Hanser, 2018). Educational intervention has been shown to aid frontline nurses, equipping them with confidence, new skills and an understanding of EIP (Batt-Rawden and Storlien, 2019). According to these authors, singing-based therapeutic caregiving has an advantage over other kinds of music intervention, because it requires no special equipment other than the singing voice. This is in line with the care of the older person ethos, which recommends non-pharmacological approaches as a complement to conventional care.

### Conclusion

Singing is a determinant of health: it makes us happier, more energetic, connects us to others, stimulates us and is a provider of joy and vitality, hence a salutogenetic activity. By facilitating opportunities for learning and practice, the staff in nursing homes can use singing as part of their art of caring, thereby enhancing environmental caring of the older person. As research from several disciplines has shown, singing has positive effects for patients, staff and the patients’ relatives, which often benefits the psychosocial working climate. Such findings indicate that nursing home employees perceive singing to have potential benefits for their patients, such as reducing uneasiness, increasing comfort, well-being and joy, promoting sleep, reducing the need for medication and lowering the potential for accidents among their patients.

## Key points for policy, practice and/or research

- If employees at nursing homes, including management, learn how to incorporate singing as an intervention into their practice, theoretical and evidence-based knowledge could be taught by teachers and other professionals from educational institutions, e.g. from Singing as care of the older person, a part of Singing Norway.
- To achieve success and lessen the barriers to the implementation of singing-based interventions in nursing homes, there needs to be a raised level of commitment, and a change in the beliefs and attitudes surrounding what constitutes quality of care and nursing, hence, engagement with and encouragement from management is important.
- Solid backing and the use of a professional music therapist or similar to support and aid this process would possibly promote even better coordination and heighten the level of engagement and motivation among actors involved for better quality of care.
- If management appreciate how singing can enhance a health-promoting workplace for its employees, it might be possible to create a stimulating, rewarding, and thriving psychosocial environment, which would benefit all actors involved.
- Singing as a method or strategy for caring of the older person ought to be included as a vital component for the enhancement of health, well-being, and quality of life for patients in nursing homes, in line with a need to build up a solid basis of positive experiences and feedback through evidence in practice.
